# Narrative Review of Patient Cancer Tissue–Derived Zebrafish Xenograft Models for Evaluating Drug Sensitivity as an Avatar Model for Clinical Application

**DOI:** 10.1002/cam4.70942

**Published:** 2025-06-26

**Authors:** Yusuke Sugino, Xin Bao, Takumi Kageyama, Sho Sekito, Shiori Miyachi, Takeshi Sasaki, Toshio Tanaka, Takahiro Inoue

**Affiliations:** ^1^ Department of Nephro‐Urologic Surgery and Andrology Mie University Graduate School of Medicine Tsu Japan; ^2^ Center for Medical Zebrafish Research Mie University Tsu Japan; ^3^ Department of Systems Pharmacology Mie University Graduate School of Medicine Tsu Japan

**Keywords:** avatar, drug sensitivity, human cancer tissues, patient‐derived xenograft model, zebrafish

## Abstract

In the pursuit of optimal medical care, treatment selection based on the molecular analysis of genomes, transcriptomes, and proteomes has been explored; however, this approach relies on data from large patient groups, resulting in limited accuracy in predicting treatment efficacy. Diseases involve complex pathological networks, requiring treatments that target multiple key molecules in these networks. Drug screening using these networks, which cannot be achieved through a gene expression analysis alone, requires animal models. Zebrafish embryos have an immature immune system, allowing for a high engraftment rate of human cancer cells transplanted within 48 h after fertilization. Consequently, the time required for engraftment is also reduced. Less than 500 human cancer cells are required for transplantation, enabling the assessment of drug efficacy from clinical samples within approximately 1 week. The cost of raising zebrafish is low, drug efficacy can be evaluated using small amounts of drugs, and their use aligns closely with animal welfare standards. This review aims to discuss the technical aspects of evaluating drug efficacy using zebrafish patient cancer tissue–derived xenograft (zPDX) models and summarize previous studies using zPDX as an avatar model for personalized medicine.

## 1 Introduction

Most medical treatments, including pharmaceutical therapy for cancers, are designed for an “average patient,” resulting in a “one‐size‐fits‐all” approach. Treatments have demonstrated efficacy in some patients; however, they have been proven ineffective in others and may occasionally produce adverse effects. Precision medicine is a highly personalized approach that enables healthcare providers to tailor treatment according to each patient's characteristics, including genome sequence, microbiome composition, health history, lifestyle, and diet. However, owing to the increasing demand for personalized medicine, treatment selection is increasingly relying on genomic, transcriptomic, and proteomic data from large patient groups [1, 2, 3]. Unfortunately, the prediction efficacy of these methods for selecting appropriate treatment is unsatisfactory [4, 5, 6]. Diseases involve complex pathophysiological networks, necessitating the use of drugs that target multiple molecules integral to these networks. Therefore, animal models that utilize these networks, which cannot be elucidated by gene expression analysis alone, are essential for drug screening. Patient‐derived xenograft mice (mPDX), established by implanting human cancer tissues into immunocompromised mice, serve as representative animal models [7, 8, 9, 10, 11, 12, 13, 14]. However, mPDX models require a long time for establishment, exhibit low engraftment rates depending on the cancer type, and incur significant costs for both the establishment and maintenance of mPDX due to the expense of using immunocompromised mice [15]. These challenges hinder the thorough evaluation of drug efficacy. Moreover, establishing mPDX models is time‐consuming, and the results of drug efficacy assessments cannot be directly applied to the patient whose cancer tissues were utilized for the mPDX establishment [7, 8, 15, 16].

Zebrafish ( *Danio rerio* ) are very popular aquarium fish and have been employed as a model for investigating developmental biology and vertebrate genetics since the 1960s. In the last few years, zebrafish have been used in various scientific fields, including human disease modeling [17, 18, 19]. In cancer research, the most significant advantage of zebrafish is their lack of an adaptive immune system during the first 12–14 days of development [20, 21, 22, 23, 24]. If human cancer cells are transplanted within 48 h after fertilization, they demonstrate a good engraftment rate. Moreover, less than 500 human cancer cells are required for transplantation, enabling the short‐term drug efficacy using a small number of clinical specimens [20, 25, 26, 27]. Zebrafish are prolific and capable of producing approximately 200 fertilized eggs per pair each week. Zebrafish larvae up to 5 days post‐fertilization are considered in vitro studies ethically [28, 29, 30]. Considering that a large number of transplants are required to evaluate drug efficacy in any given experiment to ensure high statistical power analyses, zebrafish models are more appropriate than mouse models, reducing ethical issues inherent in murine‐based experiments [20, 28, 29, 30]. Additionally, their transparency during the embryo and larval stages enables the visualization of implanted tumor cells [20]. The “Casper” strain is a pigment‐deficient one providing optically clear visualization to observe xenograft cancer cells [31, 32]. Since vivo imaging is a critical issue for detecting fluorescent xenografted cancer cells, using the strain is beneficial for high spatial resolution [31]. The Tg(*fli*:eGFP) zebrafish line with GFP‐labeled vasculature contributes largely to studies on the mechanism of tumor neovascularization and evaluations of the efficacy of antiangiogenic agents [32, 33, 34, 35]. Functional and structural homology exists between humans and zebrafish, with nearly 80% of the genes involved in human diseases shared between them [20, 24, 26]. These features facilitate cancer cell transplantation studies, the evaluation of drug efficacy, and the screening of anticancer drugs.

This review aims to summarize the technical aspects of the patient‐derived xenograft model of zebrafish (zPDX) and the application of the model in precision medicine, both currently and in the future.

## 2 Transplantation Sites for Xenografting

The yolk sac is a standard site for cancer cell transplantation, as the sac allows for easy injection of cancer cells [36, 37, 38]. This acellular compartment is composed of cholesterol, phosphatidylcholine, and triglycerides [39]. Lipids in the yolk sac are actively processed during embryogenesis to provide the fish with energy until they can feed themselves [39, 40]. Therefore, the yolk sac has been considered a favorable site for implantation as the lipids present serve as a source of nutrients for the injected human cancer cells, facilitating proliferation and growth. However, the yolk sac is not an optional site for transplantation, as the limited space may hinder tumor cell growth [41, 42]. Additionally, the viscous syncytium creates an environment that promotes cell suspension growth, which is unsuitable for the growth of anchorage‐dependent cells [41, 42]. The high lipid content may interfere with the detection of the fluorescent labels from the cancer cells transplanted into the yolk sac [25, 41]. When tumor cells are injected deep into the yolk sac, it is harder to image than tumor cells in the rather superficial perivitelline space, as described below [42]. Additionally, the use of lipophilic dyes to stain human cells derived from clinical samples can result in the retention of dead cells or debris in the yolk sac, thus producing false signals [43]. Zebrafish phagocytic cells, such as macrophages, can be stained after engulfing this debris, leading to false‐negative signals [43]. The perivitelline space, located between the periderm and yolk syncytial layer, is an avascular space that does not directly communicate with the vascular region [44]. Although most studies regarding clinical human cancer tissue xenotransplantation have selected the perivitelline space as the transplantation site (Table S1), this approach is technically more challenging than the other injection sites [45]. The ducts of Cuvier (DoCs), also known as common cardinal veins, drain into the sinus vein during embryonic development [46, 47]. Transplantation into the DoCs allows a subset of injected tumor cells to circulate throughout the zebrafish's body [47, 48]. In circulation, the tumor cells can survive, proliferate, invade, and intravasate near the caudal hematopoietic tissue, located in the tail of the embryo [48, 49, 50]. Labeled cells are traced and visualized using a fluorescence microscope. Organotopic implantation of human cancer cells has also been reported [51, 52, 53, 54, 55, 56, 57]. Marques et al. transplanted primary human pancreatic tumor cells or normal cells into the liver of zebrafish larvae and revealed that the former cells showed invasiveness and resulted in distant metastases [51]. Intracranial transplantation of glioblastoma (GBM) cells or intravitreal implantation of retinoblastoma cells into zebrafish embryos is a representative orthotopic implantation using the zebrafish model, but the technique is challenging and time‐consuming [52, 53, 55, 56, 57].

## 3 Cancer Cell Preparation

Considering the clinical flexibility of using fertilized zebrafish eggs and obtaining cancerous tissue from distant locations to evaluate drug efficacy, utilizing frozen tissue for transplantation is more practical and clinically beneficial than using fresh tissue [58, 59]. Recently, Lindahl et al. examined the cell viability of ovarian tumor tissues xenotransplanted into zebrafish, which were cut into small pieces using forceps and scalpel blades, either in fresh or cryopreserved conditions [58]. Although the cell viability was slightly reduced in cryopreserved tissues, this decrease was not practically significant; the median tumor regression observed when implanting cells isolated from cryopreserved tissues did not significantly exceed that observed from zebrafish models generated using fresh tissues [58]. However, approximately half of the studies used fresh tissues for xenotransplantation (Table S1). Ali et al. compared cell proliferation in zebrafish xenograft models derived from (1) frozen tissues enzymatically or mechanically disaggregated just before transplantation and (2) cryopreserved cell suspensions prepared after fresh tissue fragmentation, using mPDX models of non‐small cell lung cancer [59]. Results demonstrated that the former produced more viable tumors than tissues that were first disaggregated, cryopreserved as cell suspensions, and then thawed [59]. The applicability of frozen tissues for xenotransplantation may depend on the type of tissue derived and cell viability [59]. Some studies have reported alternative methods for cancer cell collection. Hua et al. selected primary cultures of non‐small cell lung cancer cells if a sufficient number of zebrafish embryos could not be prepared at the time of fresh tissue collection [60]. However, no differences were identified in the applicability of frozen tissues and primary cultured cells. Notably, they utilized malignant pleural effusion to collect cancer cells with or without cryopreservation [60]. Yin et al. developed a method to maximize the utilization of a limited quantity and size of available tissues [48]. They established a method for isolation, cryopreservation, and transient recovery of patient‐derived uveal melanoma tissues by short‐lived spheroid cultures [48]. Importantly, the spheroid culture retained the uveal melanoma properties for at least 7 d, facilitating engraftment into zebrafish and resulting in successful drug efficacy tests [48]. Regardless of whether fresh tissue, frozen tissue, or primary culture is used, the percentage of viable cells should be assessed before transplantation; only tissues with sufficient viability, verified through trypan blue staining, should be transplanted.

Human tissue samples were mostly physically disrupted with or without the use of collagenase/liberase, before xenotransplantation into zebrafish [34]. This step is crucial, as insufficient disaggregation may lead to clogging of the needle tip prepared using the borosilicate glass microcapillaries [59].

Cancer cells derived from xenotransplanted human clinical samples should be labeled for microscopic detection. In most studies, cancer cells were labeled with either Cell Tracker CM‐DiI (CM‐DiI) or carboxyfluorescein diacetate succinimidyl ester (CFSE), with the former being less cytotoxic (Table S1). CM‐DiI is a fluorescent dye and a derivative of DiI, known for its improved cellular retention and minimal cytotoxicity. DiI sometimes requires the inclusion of an osmolarity‐regulating agent or the exclusion of salt to prevent dye precipitation in the staining medium during cell labeling. By contrast, CM‐DiI circumvents these limitations, improving water solubility and enhancing staining persistence [61]. Although CM‐DiI is the most prevalent dye in zebrafish xenograft studies, DiI‐labeled particles are often detected in the caudal hematopoietic tissue in the absence of actual tumor cells [42]. Since CM‐DiI is a lipophilic dye, DiI labels dead cells and cell fragments even after engulfment by macrophages, and might even get transferred to other cells [42]. Therefore, Sturtzel et al. proposed that CellTrace Violet, a cytoplasmic stain dye, would accurately stain tumor cells, especially disseminated tumor cells, rather than CM‐DiI [42].

## 4 Zebrafish In Vivo Environment as a Host of Tumor Xenotransplantation

One limitation of the zebrafish xenograft model is the temperature constraint of the host. Zebrafish embryos are typically raised at a temperature of 28°C, while human cancer cells proliferate at a temperature of 37°C, which is the ideal temperature for cell growth. Incubating zebrafish embryos at a temperature of 32.5°C or higher from 2.5 to 96 h post‐fertilization (hpf) can lead to malformations as early as 24 hpf [62]. Although temperatures ranging from 32.5°C to 35°C can induce various embryonal malformations during the critical development period (0 hpf to 48 hpf) [63], human cancer cell xenotransplantation is typically performed at 48 hpf. Therefore, malformations do not develop in embryos incubated at 34°C or higher from 48 hpf after hatching to 3–4 days post‐injection (dpi). Importantly, the expression of various genes related to biological processes, including protein folding, oxidation–reduction processes, cellular homeostasis, and cellular component organization, can change during the regulation of heat‐stress acclimation in the zebrafish larvae [64]. These changes indirectly affect the survival or drug response of human cancer cells xenotransplanted into the zebrafish [64]. If human cancer cell proliferation decreases at lower temperatures, the efficacy of chemotherapeutic agents, such as cisplatin, which target DNA base interactions in highly proliferative cells, may be underestimated. Therefore, cancer cells must be incubated at a temperature that does not affect replication [65]. Considering these issues, an appropriate temperature balance that supports the optimal development of zebrafish embryos and the survival of human cancer cells xenotransplanted under each target and condition was established. As demonstrated in Table S1, recent studies investigating xenotransplantation of cancer cells for the evaluation of drug efficacy have utilized incubation temperatures of 34°C or 35°C for zebrafish embryos. However, this approach necessitates a reduction in the incubation period to within 4 dpi.

The adaptive immunity in zebrafish fully matures within 2–3 weeks post‐fertilization (pf), making the zebrafish larvae a potential xenograft host model for evaluating cancer drug efficacy [23, 24]. However, their highly conserved vertebrate innate immune system, including the complement, Toll‐like receptors, neutrophils, and macrophages, should be considered for successful xenotransplantation. Póvoa et al. recently reported that the crosstalk between human cancer cells and the innate immune system significantly affects cancer cell engraftment in a zebrafish xenograft model [66]. Using colon cancer cell lines, SW480 derived from the primary tumor and SW620 isolated from a lymph node metastasis isolated 6 months later, the study revealed that SW480 cells engrafted poorly, while SW620 cells exhibited high engraftment rates [66]. Gene expression analysis indicated that SW480 cells recruited neutrophils and macrophages more efficiently, while SW620 cells polarized macrophages toward an M2‐like pro‐tumoral phenotype [66]. Additionally, Gauert et al. demonstrated that the morpholino‐based transient immunosuppression of macrophages and neutrophils is necessary for optimal graft survival and growth in acute lymphoblastic leukemia [41]. Alternatively, the successful engraftment of human cancer tissue requires the chemical depletion of myeloid cells, including macrophages, using L‐clodronate during xenotransplantation [66].

## 5 Drug Administration Route for Zebrafish Human Cancer Cell Xenotransplantation Models

Compound administration is a crucial step in drug efficacy assays [44]. The traditional method for screening zebrafish larvae involves incorporating drugs directly into the embryo water, allowing the larvae to absorb the compounds without the need to administer them to each fish. 5‐Fluorouracil (5‐FU) is a water‐soluble drug. Zhai et al. examined the concentration of 5‐FU in embryos engrafted with gastric cancer cell lines using liquid chromatography–tandem mass spectrometry [67]. The 3‐dpf embryos were immersed in fresh embryo medium containing 5 mM 5‐FU, which was replaced daily for the next 3 days [67]. The concentration of 5‐FU in the embryos was increased daily following the initial treatment. The average concentrations of 5‐FU were 0.47, 0.49, and 0.71 ng per embryo at 1, 2, and 3 days post‐treatment (dpt), respectively [67]. However, the solubility, permeability, and drug are influenced by the physicochemical characteristics of each compound. This variability may cause uncertainties in the exact and effective concentrations, potentially leading to inaccurate screening results. An alternative drug administration method involves the direct inoculation of a compound either at the time of cancer cell transplantation or the day after implantation [44, 68]. In the former case, the compound concentration in the recipient zebrafish may not be standardized among individual recipient zebrafish larvae. Even if the samples are prepared with the target compound concentration, they may lack homogeneity owing to the presence of suspended cancer cells. In the latter case, the second needle insertion for drug administration may cause additional stress to the zebrafish larvae [68]. Moreover, prodrugs or compounds that function as metabolites may not be effectively metabolized. However, Van Wijk et al. identified a good correlation between the estimated absolute clearance and distribution volume in zebrafish and those in higher vertebrates [69]. Determining the toxic concentration of each compound is essential before performing the xenograft studies.

## 6 Methods of Evaluating Drug Responses in Zebrafish Human Cancer Cell Xenotransplantation Models

Most studies have employed fluorescence imaging for tumor quantification to evaluate drug responses using the zPDX model [34, 68, 70]. Tumor size analysis primarily utilizes two different techniques: (1) the use of stereo fluorescent microscope, fluorescent microscope, or inverted fluorescent microscope for image acquisition, and (2) the use of confocal microscopy and analysis of proxy of total tumor cell number via cell counting of Z‐stacks of 5 μm using a following representative formulas: “tumor size = average (Zfirst, Zmiddle, Zlast) × total number of slices/1.5” [25, 68, 71]. Lin et al. and Kowald et al. estimated the tumor size by examining the area and density of fluorescence on photomicrographs using ImageJ software at each time point, normalizing measurements to those acquired at 0 h [70, 72, 73]. Similarly, Lindhal et al. employed a nearly identical technique, but utilized a different image software (the Leica Application Suite X (LAS X) software) [58, 70, 72]. Yin et al. and Hua et al. also estimated the tumor size based on the relative measurements but normalized the result by the control tumor size measured at the same time point [48, 60]. Usai et al. measured the size of the region of interest corresponding to the DiI‐stained areas at 2 h post‐injection (hpi) and 2dpi [74]. The mean size of the tumor mass area measured at each time point was normalized to the size measured at 2 hpi [74]. Using the relative area rather than the absolute value may reduce the variability of the stained area among zebrafish owing to the differences in the sizes of xenografted tumors and the effect of lipophilic dye‐stained cell debris [74, 75]. Fior et al.'s group used normalization to respective controls [43, 76, 77], and determined the percentage of activated caspase 3 cells to estimate the percentage of apoptotic cells for evaluating drug activity at 4 dpi [34, 43, 68]. After sacrificing the zebrafish xenografts at 3 dpi, 48 h after drug application, Barroso et al. performed whole‐mount immunofluorescences, estimated the tumor size, and assessed the induction of apoptosis using Fiji/ImageJ software at a single‐cell resolution by normalization to respective control, without comparing the results with factors evaluated at 1 dpi [43, 73]. Anti‐human mitochondrial antibodies were used as markers to distinguish human cells from cellular debris or zebrafish phagocytes [43, 76, 77]. Although zebrafish embryos are transparent and suitable for many imaging applications, visualizing tissues and organs located deep within the embryo is challenging, as the embryo consists of multilayers of tissues [46, 78]. The heart and digestive system, due to the slight absorbance of light by each tissue, which may obscure the target region [46, 78]. The yolk is even more difficult for light to pass through, so tissues located between the yolk and other tissue layers are the most challenging to image [46, 78]. Alternatively, cryosectioning, vibratome, or microtome sectioning are available with or without fixation [79]. However, these methods are highly sensitive to the specific angle and region of the cut, and landmark features are necessary to determine where each section was obtained from the whole zebrafish [78, 80]. Moreover, acquiring all the necessary sections to gather information on the entire tissue structure for the analysis might be challenging [78]. Wu et al. sacrificed and dissociated 10 embryos from the control and treatment groups into a single‐cell suspension before and after drug exposure at 2 dpt (3 dpi) [81]. The number of CM‐DiI‐labeled cells was counted to confirm the successful engraftment and proliferation of the cells in the zebrafish [81]. Additionally, the cells were co‐stained with DRAQ5 nuclear stain to confirm the enumeration of human cancer cells and to exclude non‐specific staining of tissue debris [81]. They observed that fluorescent‐labeled cells were distributed in a three‐dimensional manner throughout the embryo after injection [81]. Thus, the measurement of photomicrographs based on fluorescence density may not accurately reflect the actual number of cells [81]. Additionally, the red fluorescent density of CM‐DiI‐labeled cells tends to fade as the cells divide, indicating that the fluorescent density does not enhance cell proliferation [81]. Bentley et al. transplanted primary patient–derived T‐cell acute lymphoblastic leukemia cells into zebrafish and treated them with rapamycin, triciribine, and compound E at 96 hpi [82]. At 48 hpt, the zebrafish were sacrificed and dissociated. Cell viability was assessed by counting the cells positive for Promyelocytic leukemia protein (PML) bodies stained with human anti‐PML antibodies [82]. In a manuscript by Gauert et al., pooled zebrafish embryos transplanted with human leukemia cells were sacrificed and dissociated for immunostaining and flow cytometry analysis [41]. Gauert et al. used an anti‐human CD19 antibody, 7‐aminoactinomycin D viability staining solution, and fluorescein isothiocyanate‐conjugated annexin A5 to assess cell viability [41]. Al‐Samadi et al. evaluated a patient‐derived tongue tumor cell numbers in zebrafish larvae by performing a droplet digital polymerase chain reaction (ddPCR) using human‐specific glyceraldehyde 3‐phosphate dehydrogenase primers. They reported that the method was quicker and easier than traditional imaging analysis [83]. However, due to the limited availability of ddPCR and comparable results were evaluated by quantitative PCR, they proposed using quantitative PCR for clinical applications [83]. Quantitative PCR is easily adopted but if the number of the target human epithelial cancer cells is low, it cannot detect any epithelial cancer cell markers such as cytokeratin. Various estimation methods for evaluating drug efficacy have been reported. When analyzing tumor size using one method, the results should be confirmed using additional techniques, such as measuring apoptosis or evaluating cell proliferation markers, like Ki 67.

## 7 Zebrafish Human Cancer Cell Xenotransplantation Models as a Potential Avatar Model for Drug Administration

Fior et al. demonstrated a correlation between patients and their zPDX in terms of sensitivity to chemotherapeutic agents (e.g., folinic acid, 5‐FU, and oxaliplatin [FOLFOX] in the treatment of colorectal cancer [CRC]) [34]. De Almedia et al. compared the bevacizumab responses of two patients with colorectal cancer (CRC) using matched zPDX models. They revealed that resistance to bevacizumab treatment in the zPDX models reflected the patients' outcomes, indicating the potential of zPDX models as avatar models for investigating drug treatment outcomes [68]. Interestingly, the two samples collected before transplantation comprised a mixture of tumor cells and stroma, such as immune cells [68]. Similar issues have been reported by Di Franco et al., Costa et al., and Barroso et al., who detected non‐tumor cells in zebrafish before and after transplantation [43, 77, 84]. These studies suggested that zPDX models may potentially offer advantages over tumor genetic analysis or organoid culture, as they can evaluate not only tumor cells but also the tumor microenvironment. Ali et al. transplanted 39 non‐small cell lung cancer mPDX models into zebrafish larvae to evaluate paclitaxel and erlotinib efficacy and the metastatic capacity of tumor cells [59]. They revealed that zPDX models accurately predicted the treatment outcomes and metastatic characteristics of patients compared with their parental mPDX models [59]. Furthermore, they successfully transplanted four cases of non‐small cell lung cancer tissues into zebrafish larvae and characterized their metastatic capacity and response to paclitaxel and erlotinib [59]. As zPDX models cannot expand tumor tissues or fully characterize the biological aspects of the tumors, the authors suggested that combining mPDX and zPDX models could potentially create synergetic preclinical models that address the drawbacks of each model [59]. Ai et al. expanded primary GBM cells in a short‐term culture in vitro, and after labeling the cells with CFSE or with infection with lentivirus containing a CMV driving mCherry gene, they were injected into the zebrafish brain [85]. Five primary GBM tissues were surgically resected and transplanted into the zebrafish brain to check sensitivity for temozolomide (TMZ) [85]. Three cases were sensitive, and two cases were refractory to TMZ in zPDX models, and the results mirrored their clinical outcomes by TMZ treatment, showing that the orthotopic GBM transplantation zPDX model might be informative to determine TMZ treatment for GBM [85].

Kowald et al. established a zPDX model to evaluate the efficacy of immune‐stimulated Bacillus Calmette‐Guerin (BCG) therapy in bladder cancer [72]. They demonstrated the importance of physical contact between viable BCG bacteria and tumor cells, as co‐injection of pre‐washed BCG and bladder cancer cells was more effective than the intravenous injection of pre‐washed or heat‐inactivated BCG [72]. Their zPDX model was utilized to estimate the anti‐tumor immune response elicited by BCG through the stimulation of peripheral blood mononuclear cells and the activation of cytotoxic natural killer cell responses [72].

DiFranco et al. conducted a co‐clinical trial to evaluate the use of the zPDX model in predicting the response to several chemotherapy regimens in patients with CRC [75]. Eight had available data on their response to first‐line chemotherapy and the zPDX chemosensitivity profile of their tumor during the follow‐up period [75]. Among the eight patients, five experienced a partial response, while three exhibited stable disease in response to chemotherapy. The results demonstrated a concordance rate of 75% (6/8 cases) between the clinical response and the efficacy of chemotherapy reported in the zPDXs [75]. Costa et al. presented the results of a co‐clinical study, which compared the chemotherapy clinical responses of 55 CRC patients with the corresponding zPDX models [76]. Among these patients, the zPDX models successfully predicted the outcomes of 50 patients, distinguishing between non‐progression/stable and progressive disease following systemic therapy [76]. They insisted that the patients' tumor stage, metastatic potential, and apoptotic changes analyzed in the zPDX model were the most important predictors of clinical response [76]. By integrating the three variables into a decision tree algorithm, the zPDX models achieved a positive predictive value of 91% and a negative predictive value of 90%. This finding suggests that the zPDX model is a potential and efficient tool for guiding clinical decisions [76].

Lindahl et al. examined ovarian cancer zPDX models [58]. Among the nine patients evaluated for sensitivity to carboplatin, two progressed within the 2‐year follow‐up period [58]. These patients were included in five zPDX models that exhibited a poor response to carboplatin [58]. Among the seven patients who did not progress within the 2‐year follow‐up period, four zPDX models who demonstrated a response to carboplatin were included among those generated from the seven patients with stable disease [58]. Although these results demonstrated the utility of the zPDX model in clinical decision‐making, the correlation between short‐term drug sensitivity and long‐term clinical outcomes was not optimal [58]. The discrepancy in clinical drug resistance rates among patients displaying drug sensitivity in the zPDX models might be attributed to tumor heterogeneity, regrowth of “cancer stem cells”, and other factors that are not evaluated by short‐term zPDX assays [58]. However, the clinical drug response among those demonstrating resistance in the zPDX models could be explained by the potentially complete removal of the tumors during ovarian cancer debulking surgery, the ability of the patient's immune system to eradicate the remaining tumors after surgery, the enhanced efficacy of other drugs used in combination therapy, drug–drug interactions, or other unknown factors that are difficult to evaluate using zPDX models [58].

## 8 Future Perspectives of Patient‐Derived Zebrafish as an Avatar Model in Clinical Oncology for Estimating Drug Sensitivity

**FIGURE 1:**
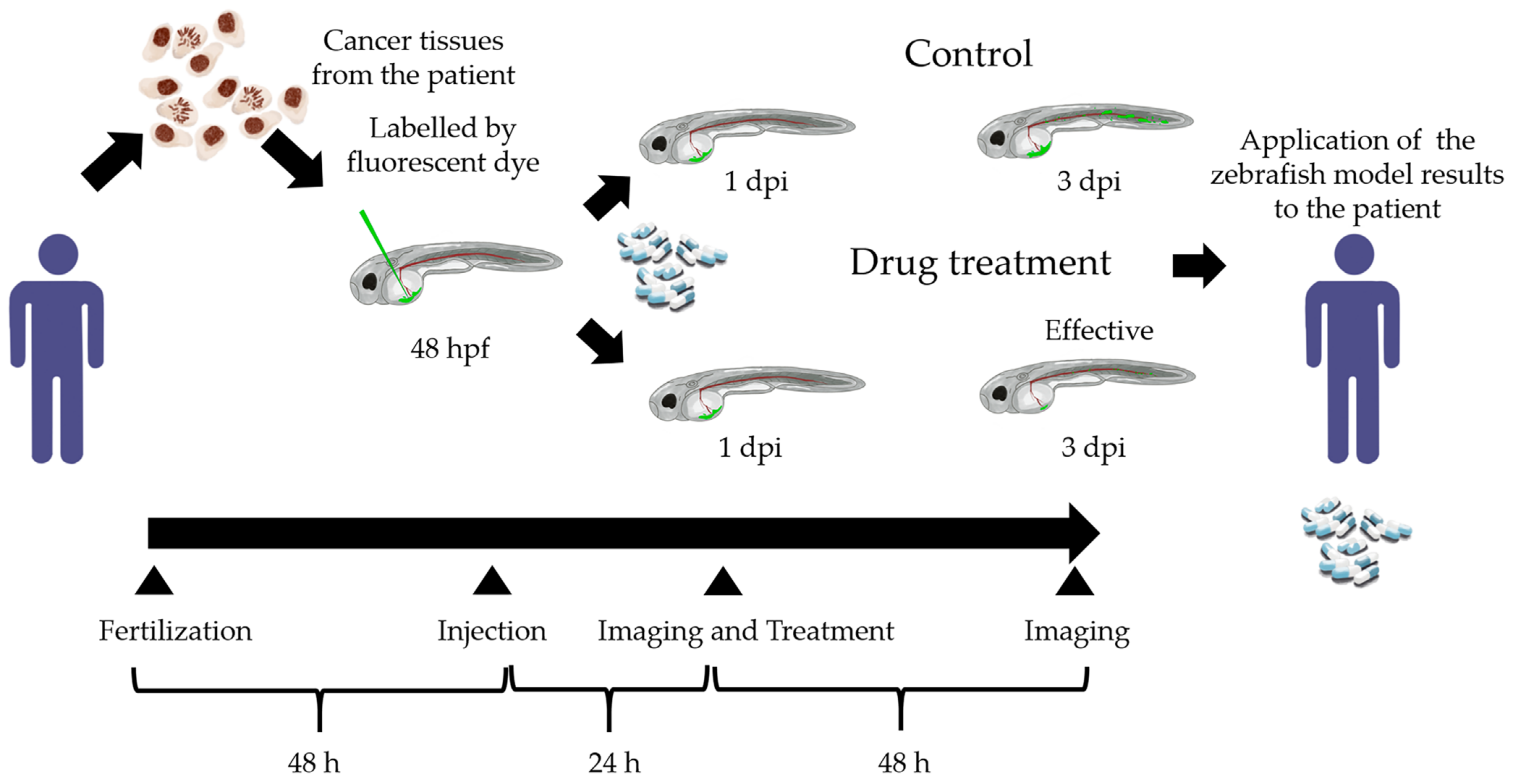
Representative method of applying patient cancer tissue–derived zebrafish xenograft models as an avatar model. Cancer tissues are extracted from the patient and prepared for engraftment. Fluorescent‐labeled cancer cells are transplanted at 48 h post‐fertilization (hpf), and their images are taken at 1‐day post‐injection (dpi) and 3 dpi, 48 h after drug treatment. The fold changes in the areas (3 dpi/1 dpi) above a preset threshold of fluorescent dye are calculated to estimate cancer growth/reduction. The results are applied to determine whether the target drug is beneficial for the treatment of the patient.

The patient‐derived zebrafish model, which enables next‐generation personalized cancer medicine, is a technology that involves transplanting the patient's cancer tissues into zebrafish and observing their sensitivity to select optimal therapeutic drugs. In order to apply this model to clinical fields, efficient and stable methodologies of breeding and husbandry of zebrafish are essential for the zPDX model [71, 86, 87]. Evaluating the optimal methods of administration of various drugs, their metabolism, and resulting metabolites in zebrafish is also essential. Patient‐derived cells lack intrinsic fluorescent proteins for imaging analysis, requiring labeling of the cells. Fluorescent dyes should selectively stain live cells while leaving dead cells uncolored. Moreover, the stained cells must have a clear outline for accurate estimation. Although various imaging setups have been employed, a potential and widely acceptable automated workflow should be established for image acquisition of xenografted zebrafish, tumor cell detection, and tumor size quantification utilizing a deep learning‐based algorithm based on automatic segmentation [42, 88]. The standardization of these detailed techniques will enable the zPDX model to become applicable to clinical trials (Figure 1).

## 9 Conclusions

Zebrafish xenografts offer a cost‐effective and time‐efficient tool for estimating drug efficacy, while also providing a statistically sufficient sample size for clinical application, which is not obtainable with mammalian models. However, before applying this avatar model to the clinical cancer field, randomized clinical trials, which prove benefit for selecting candidate drug treatment according to the zebrafish xenograft drug efficacy, are mandatory for zPDX clinical implementation in the future. Although a zebrafish xenograft model has drawbacks and requires further refinement, the model will likely play an important role in facilitating personalized treatment for each patient.

## Author Contributions

T.I. conceived the project. Y.S., B.X., and T.I. wrote the manuscript with input from all the authors. All the authors contributed to editing and reviewing the manuscript.

## Ethics Statement

The authors have nothing to report.

## Conflicts of Interest

The authors declare no conflicts of interest.

## Supporting information


Table S1.

## Data Availability

The authors have nothing to report.
